# Supplementary material for: Pyruvate Dehydrogenase Kinase 1 inhibition mediated oxidative phosphorylation enhancement in cartilage promotes osteoarthritis progression
Source: BMC Musculoskelet Disord. 2023 Jul 20;24:597. doi: 10.1186/s12891-023-06585-6 (PMC10357736; doi:10.1186/s12891-023-06585-6)
Supplement: Supplementary file 1 — Additional file 1. [file 12891_2023_6585_MOESM1_ESM.zip › BP.Gsea.1653623667859/GOBP_MEMBRANE_INVAGINATION.html]

Details for gene set GOBP\_MEMBRANE\_INVAGINATION[GSEA]

|  || Dataset | 55457\_collapsed\_to\_symbols.cls.cls #OA\_versus\_normal.cls.cls #OA\_versus\_normal\_repos |
| Phenotype | cls.cls#OA\_versus\_normal\_repos |
| Upregulated in class | OA |
| GeneSet | GOBP\_MEMBRANE\_INVAGINATION |
| Enrichment Score (ES) | 0.39788598 |
| Normalized Enrichment Score (NES) | 1.7736286 |
| Nominal p-value | 0.002386635 |
| FDR q-value | 0.09383047 |
| FWER p-Value | 0.993 |
Table: GSEA Results Summary

  

Fig 1: Enrichment plot: GOBP\_MEMBRANE\_INVAGINATION      
 Profile of the Running ES Score & Positions of GeneSet Members on the Rank Ordered List

  

| SYMBOL | TITLE | RANK IN GENE LIST | RANK METRIC SCORE | RUNNING ES | CORE ENRICHMENT || 1 | TREM2 | triggering receptor expressed on myeloid cells 2 [Source:HGNC Symbol;Acc:HGNC:17761] | 342 | 0.726 | 0.0019 | Yes |
| 2 | ELMO1 | engulfment and cell motility 1 [Source:HGNC Symbol;Acc:HGNC:16286] | 370 | 0.718 | 0.0281 | Yes |
| 3 | CHMP4A | charged multivesicular body protein 4A [Source:HGNC Symbol;Acc:HGNC:20274] | 526 | 0.649 | 0.0415 | Yes |
| 4 | APPL2 | "adaptor protein, phosphotyrosine interacting with PH domain and leucine zipper 2 [Source:HGNC Symbol;Acc:HGNC:18242]" | 586 | 0.628 | 0.0616 | Yes |
| 5 | IGHG1 | immunoglobulin heavy constant gamma 1 (G1m marker) [Source:HGNC Symbol;Acc:HGNC:5525] | 607 | 0.621 | 0.0845 | Yes |
| 6 | FCGR1A | Fc fragment of IgG receptor Ia [Source:HGNC Symbol;Acc:HGNC:3613] | 633 | 0.610 | 0.1066 | Yes |
| 7 | ADGRB1 | adhesion G protein-coupled receptor B1 [Source:HGNC Symbol;Acc:HGNC:943] | 655 | 0.605 | 0.1288 | Yes |
| 8 | PLCG2 | phospholipase C gamma 2 [Source:HGNC Symbol;Acc:HGNC:9066] | 725 | 0.588 | 0.1465 | Yes |
| 9 | CD300A | CD300a molecule [Source:HGNC Symbol;Acc:HGNC:19319] | 730 | 0.587 | 0.1693 | Yes |
| 10 | ITGB2 | integrin subunit beta 2 [Source:HGNC Symbol;Acc:HGNC:6155] | 827 | 0.562 | 0.1839 | Yes |
| 11 | IGHV3-72 | immunoglobulin heavy variable 3-72 [Source:HGNC Symbol;Acc:HGNC:5622] | 1062 | 0.504 | 0.1855 | Yes |
| 12 | TRDC | T cell receptor delta constant [Source:HGNC Symbol;Acc:HGNC:12253] | 1100 | 0.496 | 0.2021 | Yes |
| 13 | IGLC2 | immunoglobulin lambda constant 2 [Source:HGNC Symbol;Acc:HGNC:5856] | 1210 | 0.474 | 0.2123 | Yes |
| 14 | CHMP2A | charged multivesicular body protein 2A [Source:HGNC Symbol;Acc:HGNC:30216] | 1269 | 0.464 | 0.2260 | Yes |
| 15 | AURKB | aurora kinase B [Source:HGNC Symbol;Acc:HGNC:11390] | 1407 | 0.442 | 0.2327 | Yes |
| 16 | IGHA1 | immunoglobulin heavy constant alpha 1 [Source:HGNC Symbol;Acc:HGNC:5478] | 1426 | 0.439 | 0.2486 | Yes |
| 17 | TRBC1 | T cell receptor beta constant 1 [Source:HGNC Symbol;Acc:HGNC:12156] | 1458 | 0.435 | 0.2633 | Yes |
| 18 | AIF1 | allograft inflammatory factor 1 [Source:HGNC Symbol;Acc:HGNC:352] | 1462 | 0.434 | 0.2802 | Yes |
| 19 | IGKC | immunoglobulin kappa constant [Source:HGNC Symbol;Acc:HGNC:5716] | 1477 | 0.432 | 0.2961 | Yes |
| 20 | IGHV3-33 | immunoglobulin heavy variable 3-33 [Source:HGNC Symbol;Acc:HGNC:5596] | 1511 | 0.427 | 0.3103 | Yes |
| 21 | CLCN3 | chloride voltage-gated channel 3 [Source:HGNC Symbol;Acc:HGNC:2021] | 1535 | 0.423 | 0.3252 | Yes |
| 22 | ARHGAP25 | Rho GTPase activating protein 25 [Source:HGNC Symbol;Acc:HGNC:28951] | 1900 | 0.373 | 0.3115 | Yes |
| 23 | FCGR2B | Fc fragment of IgG receptor IIb [Source:HGNC Symbol;Acc:HGNC:3618] | 2016 | 0.360 | 0.3167 | Yes |
| 24 | RAB31 | "RAB31, member RAS oncogene family [Source:HGNC Symbol;Acc:HGNC:9771]" | 2232 | 0.335 | 0.3131 | Yes |
| 25 | IGHM | immunoglobulin heavy constant mu [Source:HGNC Symbol;Acc:HGNC:5541] | 2241 | 0.335 | 0.3256 | Yes |
| 26 | GULP1 | GULP PTB domain containing engulfment adaptor 1 [Source:HGNC Symbol;Acc:HGNC:18649] | 2323 | 0.324 | 0.3320 | Yes |
| 27 | IGHV3-21 | immunoglobulin heavy variable 3-21 [Source:HGNC Symbol;Acc:HGNC:5586] | 2404 | 0.314 | 0.3382 | Yes |
| 28 | RHOG | ras homolog family member G [Source:HGNC Symbol;Acc:HGNC:672] | 2437 | 0.310 | 0.3479 | Yes |
| 29 | IGHV1-69 | immunoglobulin heavy variable 1-69 [Source:HGNC Symbol;Acc:HGNC:5558] | 2461 | 0.307 | 0.3582 | Yes |
| 30 | NCKAP1L | NCK associated protein 1 like [Source:HGNC Symbol;Acc:HGNC:4862] | 2560 | 0.296 | 0.3622 | Yes |
| 31 | RHOH | ras homolog family member H [Source:HGNC Symbol;Acc:HGNC:686] | 2680 | 0.282 | 0.3640 | Yes |
| 32 | IGHV4-61 | immunoglobulin heavy variable 4-61 [Source:HGNC Symbol;Acc:HGNC:5655] | 2722 | 0.278 | 0.3717 | Yes |
| 33 | C3 | complement C3 [Source:HGNC Symbol;Acc:HGNC:1318] | 2764 | 0.273 | 0.3793 | Yes |
| 34 | IGHV3-7 | immunoglobulin heavy variable 3-7 [Source:HGNC Symbol;Acc:HGNC:5620] | 2798 | 0.270 | 0.3873 | Yes |
| 35 | XKR8 | XK related 8 [Source:HGNC Symbol;Acc:HGNC:25508] | 2884 | 0.262 | 0.3910 | Yes |
| 36 | IGHV3-73 | immunoglobulin heavy variable 3-73 [Source:HGNC Symbol;Acc:HGNC:5623] | 3082 | 0.241 | 0.3852 | Yes |
| 37 | STAP1 | signal transducing adaptor family member 1 [Source:HGNC Symbol;Acc:HGNC:24133] | 3213 | 0.229 | 0.3840 | Yes |
| 38 | VAMP7 | vesicle associated membrane protein 7 [Source:HGNC Symbol;Acc:HGNC:11486] | 3380 | 0.214 | 0.3795 | Yes |
| 39 | TRBC2 | T cell receptor beta constant 2 [Source:HGNC Symbol;Acc:HGNC:12157] | 3401 | 0.211 | 0.3862 | Yes |
| 40 | IGHV3-23 | immunoglobulin heavy variable 3-23 [Source:HGNC Symbol;Acc:HGNC:5588] | 3449 | 0.207 | 0.3907 | Yes |
| 41 | SNX3 | sorting nexin 3 [Source:HGNC Symbol;Acc:HGNC:11174] | 3469 | 0.205 | 0.3973 | Yes |
| 42 | RHOBTB1 | Rho related BTB domain containing 1 [Source:HGNC Symbol;Acc:HGNC:18738] | 3562 | 0.197 | 0.3979 | Yes |
| 43 | ALKBH4 | "alkB homolog 4, lysine demethylase [Source:HGNC Symbol;Acc:HGNC:21900]" | 4264 | 0.131 | 0.3484 | No |
| 44 | BIN2 | bridging integrator 2 [Source:HGNC Symbol;Acc:HGNC:1053] | 4390 | 0.122 | 0.3434 | No |
| 45 | IGHV4-34 | immunoglobulin heavy variable 4-34 [Source:HGNC Symbol;Acc:HGNC:5650] | 4746 | 0.091 | 0.3193 | No |
| 46 | HAVCR1 | hepatitis A virus cellular receptor 1 [Source:HGNC Symbol;Acc:HGNC:17866] | 4902 | 0.080 | 0.3104 | No |
| 47 | DOCK1 | dedicator of cytokinesis 1 [Source:HGNC Symbol;Acc:HGNC:2987] | 4998 | 0.073 | 0.3059 | No |
| 48 | MARCO | macrophage receptor with collagenous structure [Source:HGNC Symbol;Acc:HGNC:6895] | 5007 | 0.072 | 0.3081 | No |
| 49 | ITGAM | integrin subunit alpha M [Source:HGNC Symbol;Acc:HGNC:6149] | 5820 | 0.007 | 0.2450 | No |
| 50 | RAC2 | Rac family small GTPase 2 [Source:HGNC Symbol;Acc:HGNC:9802] | 6098 | -0.013 | 0.2239 | No |
| 51 | ARHGAP12 | Rho GTPase activating protein 12 [Source:HGNC Symbol;Acc:HGNC:16348] | 6099 | -0.013 | 0.2244 | No |
| 52 | IGHV5-51 | immunoglobulin heavy variable 5-51 [Source:HGNC Symbol;Acc:HGNC:5659] | 6399 | -0.035 | 0.2025 | No |
| 53 | IGHV3-20 | immunoglobulin heavy variable 3-20 [Source:HGNC Symbol;Acc:HGNC:5585] | 6859 | -0.068 | 0.1694 | No |
| 54 | CLEC7A | C-type lectin domain containing 7A [Source:HGNC Symbol;Acc:HGNC:14558] | 7090 | -0.087 | 0.1548 | No |
| 55 | ARF1 | ADP ribosylation factor 1 [Source:HGNC Symbol;Acc:HGNC:652] | 7152 | -0.092 | 0.1537 | No |
| 56 | RAC1 | Rac family small GTPase 1 [Source:HGNC Symbol;Acc:HGNC:9801] | 7192 | -0.096 | 0.1544 | No |
| 57 | IGLL1 | immunoglobulin lambda like polypeptide 1 [Source:HGNC Symbol;Acc:HGNC:5870] | 7544 | -0.122 | 0.1319 | No |
| 58 | MFGE8 | milk fat globule EGF and factor V/VIII domain containing [Source:HGNC Symbol;Acc:HGNC:7036] | 7756 | -0.138 | 0.1208 | No |
| 59 | ITGA2 | integrin subunit alpha 2 [Source:HGNC Symbol;Acc:HGNC:6137] | 7857 | -0.145 | 0.1187 | No |
| 60 | CD36 | CD36 molecule [Source:HGNC Symbol;Acc:HGNC:1663] | 7985 | -0.154 | 0.1149 | No |
| 61 | MSR1 | macrophage scavenger receptor 1 [Source:HGNC Symbol;Acc:HGNC:7376] | 8083 | -0.162 | 0.1137 | No |
| 62 | MYH9 | myosin heavy chain 9 [Source:HGNC Symbol;Acc:HGNC:7579] | 8463 | -0.191 | 0.0916 | No |
| 63 | GSN | gelsolin [Source:HGNC Symbol;Acc:HGNC:4620] | 8507 | -0.194 | 0.0959 | No |
| 64 | RHOA | ras homolog family member A [Source:HGNC Symbol;Acc:HGNC:667] | 8601 | -0.201 | 0.0966 | No |
| 65 | F2RL1 | F2R like trypsin receptor 1 [Source:HGNC Symbol;Acc:HGNC:3538] | 8631 | -0.203 | 0.1023 | No |
| 66 | BECN1 | beclin 1 [Source:HGNC Symbol;Acc:HGNC:1034] | 8644 | -0.204 | 0.1094 | No |
| 67 | ABCA7 | ATP binding cassette subfamily A member 7 [Source:HGNC Symbol;Acc:HGNC:37] | 8856 | -0.222 | 0.1017 | No |
| 68 | IGHD | immunoglobulin heavy constant delta [Source:HGNC Symbol;Acc:HGNC:5480] | 9396 | -0.263 | 0.0700 | No |
| 69 | ABCA1 | ATP binding cassette subfamily A member 1 [Source:HGNC Symbol;Acc:HGNC:29] | 9874 | -0.309 | 0.0450 | No |
| 70 | SH3BP1 | SH3 domain binding protein 1 [Source:HGNC Symbol;Acc:HGNC:10824] | 10016 | -0.323 | 0.0467 | No |
| 71 | FNBP1L | formin binding protein 1 like [Source:HGNC Symbol;Acc:HGNC:20851] | 10082 | -0.329 | 0.0545 | No |
| 72 | HGS | hepatocyte growth factor-regulated tyrosine kinase substrate [Source:HGNC Symbol;Acc:HGNC:4897] | 10315 | -0.352 | 0.0503 | No |
| 73 | IGHE | immunoglobulin heavy constant epsilon [Source:HGNC Symbol;Acc:HGNC:5522] | 10676 | -0.390 | 0.0376 | No |
| 74 | GATA2 | GATA binding protein 2 [Source:HGNC Symbol;Acc:HGNC:4171] | 11058 | -0.439 | 0.0252 | No |
| 75 | RHOBTB2 | Rho related BTB domain containing 2 [Source:HGNC Symbol;Acc:HGNC:18756] | 11303 | -0.471 | 0.0247 | No |
| 76 | ALOX15 | arachidonate 15-lipoxygenase [Source:HGNC Symbol;Acc:HGNC:433] | 11405 | -0.488 | 0.0360 | No |
| 77 | THBS1 | thrombospondin 1 [Source:HGNC Symbol;Acc:HGNC:11785] | 11498 | -0.501 | 0.0485 | No |
| 78 | SMURF1 | SMAD specific E3 ubiquitin protein ligase 1 [Source:HGNC Symbol;Acc:HGNC:16807] | 12344 | -0.688 | 0.0097 | No |
| 79 | CDC42 | cell division cycle 42 [Source:HGNC Symbol;Acc:HGNC:1736] | 12681 | -0.852 | 0.0171 | No |
Table: GSEA details [plain text format]

  

Fig 2: GOBP\_MEMBRANE\_INVAGINATION      
 Blue-Pink O' Gram in the Space of the Analyzed GeneSet

  

Fig 3: GOBP\_MEMBRANE\_INVAGINATION: Random ES distribution      
 Gene set null distribution of ES for **GOBP\_MEMBRANE\_INVAGINATION**
